# Protocol for PARA-PET: a trial of [18F]fluorocholine PET/CT versus conventional imaging for primary hyperparathyroidism with randomised order of blinded imaging assessment

**DOI:** 10.1136/bmjopen-2026-122455

**Published:** 2026-10-29

**Authors:** Elizabeth Wootton, Clement Wong, Peter Donovan, Donald S A Mcleod, M Grossmann, Samuel Kyle, Louise Campbell, Anita Pelecanos, Louisa G Collins, Roslyn Francis, David A Pattison

**Affiliations:** 1 Endocrinology and Diabetes, Royal Brisbane and Women’s Hospital, Herston, Queensland, Australia; 2 Faculty of Medicine, The University of Queensland, Brisbane, Queensland, Australia; 3 Endocrine Surgery, Royal Brisbane and Women’s Hospital, Herston, Queensland, Australia; 4 QIMR Berghofer Medical Research Institute, Herston, Queensland, Australia; 5 Faculty of Medicine, University of Melbourne, Melbourne, Victoria, Australia; 6 Endocrinology and Diabetes, Austin Health, Melbourne, Victoria, Australia; 7 Medical Imaging, Royal Brisbane and Women’s Hospital, Herston, Queensland, Australia; 8 Nuclear Medicine and PET Services, Royal Brisbane and Women’s Hospital, Herston, Queensland, Australia; 9 Statistics, QIMR Berghofer Medical Research Institute, Herston, Queensland, Australia; 10 Cancer Council Queensland, Brisbane, Queensland, Australia

**Keywords:** General endocrinology, NUCLEAR MEDICINE, RADIOLOGY & IMAGING

## Abstract

**Introduction:**

Primary hyperparathyroidism is a common endocrine condition due to inappropriate parathyroid hormone production from one or more parathyroid glands. Surgery is curative. Imaging performed preoperatively aims to accurately localise the responsible gland(s) to facilitate minimally invasive surgery. Current imaging techniques with neck ultrasound (US), sestamibi single-photon emission tomography (SPECT)/CT and four-dimensional CT (4DCT) are imperfect. [^18^F]Fluorocholine (FCH) PET/CT is a novel modality offering several advantages, including improved accuracy, lower radiation exposure and shorter imaging time. This protocol describes PARA-PET, a non-inferiority trial designed to compare FCH PET/CT to conventional imaging for primary hyperparathyroidism.

**Methods and analysis:**

134 patients with primary hyperparathyroidism referred for surgery will receive FCH PET/CT in addition to conventional imaging (sestamibi SPECT/CT and 4DCT), as well as neck US for operative planning. All imaging techniques will be reviewed at a monthly multidisciplinary meeting, attended by representatives from Endocrinology, Endocrine Surgery, Nuclear Medicine and Radiology. Participants will be randomised 1:1 to the order in which the diagnostic strategy will be reviewed first, with interpretation blinded to alternative imaging arm results. The intended surgical approach will be prospectively documented. Clinical outcomes will be followed up at 1 and 6 months post-surgery. Additional analyses will include accuracy, patient satisfaction, cost effectiveness and environmental impact of each imaging technique.

**Ethics and dissemination:**

The study has received ethical approval from the Metro North Health Research Ethics Committee (Project ID: 113012; HREC/2025/MNH/113012). Participants will be sent a letter summarising the study results and inviting them to contact the research team with any questions. The trial will be published in an international peer-reviewed journal. Abstracts will also be submitted to relevant national and international endocrine and nuclear medicine scientific meetings.

**Trial registration:**

ANZCTR ACTRN12625000509460.

**STRENGTHS AND LIMITATIONS OF THIS STUDY**

- This study will be a large prospective trial assessing the management impact of [^18^F]fluorocholine PET/CT compared with sestamibi single-photon emission tomography/CT and four-dimensional CT in individuals with primary hyperparathyroidism, with randomised order of blinded imaging review.
- Performing all imaging modalities within the same individual facilitates direct comparison of accuracy.
- Secondary outcomes exploring patient satisfaction, cost-effectiveness and environmental impact of different parathyroid imaging strategies will provide important insights to impact healthcare practices and resource utilisation.
- Limitations include the review of all imaging techniques prior to parathyroid surgery which precludes the attribution of any difference in clinical outcomes solely to one imaging strategy.

## Introduction

 Primary hyperparathyroidism (PHPT) is a common endocrine disease that results in overproduction of parathyroid hormone (PTH), leading to considerable morbidity if untreated. In approximately 85% of cases PHPT is due to a single parathyroid adenoma.^1 2^ Surgical resection of the aberrant gland is the only curative treatment, with accurate preoperative localisation of the hyperfunctioning gland necessary to enable a minimally invasive or targeted surgical approach (MITP).^3^ Without accurate preoperative localisation, bilateral neck exploration (BNE) is required, with increased risk of recurrent laryngeal nerve damage, postoperative hypoparathyroidism and neck scarring.

The current standard of care (‘conventional imaging’) in Australia is to undertake multimodal imaging modalities with neck ultrasound (US), [^99m^Tc]Sestamibi (sestamibi) single-photon emission computed tomography (SPECT)/CT and four-dimensional computed tomography (4DCT). Accuracy rates of localisation are imperfect, approximating 57% for neck US, 65–70% for sestamibi SPECT/CT and 80–87% for 4DCT.^4–6^ [^18^F]Fluorocholine (FCH) PET/CT is a novel imaging technique increasingly recognised as the most accurate modality for parathyroid adenoma localisation, with reported sensitivity approaching 95%.^7^

Additional advantages of FCH PET/CT in comparison to conventional imaging modalities include:

1. More accurate localisation than sestamibi SPECT/CTdue to higher spatial resolution of PET imaging, which can be especially beneficial in cases of multi-glandular involvement or in cases requiring revision surgery.^8^
2. Substantially shorter imaging time (approximately 1 hour) compared with sestamibi SPECT/CT (up to 4 hours).^8^
3. Lower radiation doses compared with sestamibi SPECT/CT and 4DCT.^9^
4. Avoiding complications of iodinated-contrast administration associated with 4DCT.^10^

FCH PET/CT is currently considered by the European Association of Nuclear Medicine (EANM) as an alternative first-line imaging method for PHPT.^8^ The recent position statement of the Endocrine Society of Australia, the Australian & New Zealand Endocrine Surgeons and the Australian & New Zealand Bone and Mineral Society on PHPT highlights FCH PET/CT as showing promise but does not make a specific recommendation on its use due to limited data.^11^ There are also no published evaluations of environmental impact, patient experience or cost-effectiveness in the Australian setting.

## Objectives

### Aims

1. To evaluate whether FCH PET/CT is non-inferior to conventional imaging (sestamibi SPECT/CT and 4DCT) in management impact of PHPT, defined by change in intended surgical approach from BNE to MITP.
2. To compare the proportion of each of the four imaging modalities (FCH PET/CT, neck US, sestamibi SPECT/CT and 4DCT) to accurately localise parathyroid adenoma(s) in participants achieving cure after surgery.
3. In participants whose first-line imaging failed to localise a parathyroid adenoma, to compare the proportion with a change in management impact after second-line imaging.
4. To analyse the cost-effectiveness, radiation exposure and carbon footprint of each diagnostic strategy.
5. To explore the patient experience with FCH PET/CT compared with conventional imaging, using quantitative and qualitative responses.
6. To assess inter-reporter agreement of interpretation of each diagnostic imaging strategy.

### Hypotheses

In patients with PTH-dependent hypercalcaemia referred for surgery, it is hypothesised that FCH PET/CT will, when compared with conventional imaging (sestamibi SPECT/CT and 4DCT):

- Have a non-inferior proportion of change in intended surgical approach from BNE to MITP.
- Have a higher proportion of accurate parathyroid adenoma localisation in participants achieving post-operative cure.
- Have a higher proportion of change in management after second-line imaging, in those whose first-line imaging failed to localise a parathyroid adenoma.
- Be cost-effective or cost-saving to the Australian health system.
- Reduce the radiation exposure and environmental impact of work up for PHPT.
- Increase patient satisfaction with the imaging work-up for parathyroid surgery.
- Have a similar inter-reporter agreement of interpretation.

## Trial design

We will conduct a single-centre prospective non-inferiority trial of FCH PET/CT compared with conventional imaging in patients requiring surgical management of PTH-dependent hypercalcaemia (figure 1). All participants will receive both imaging strategies with randomisation determining the order of blinded imaging review.

**Figure 1:**
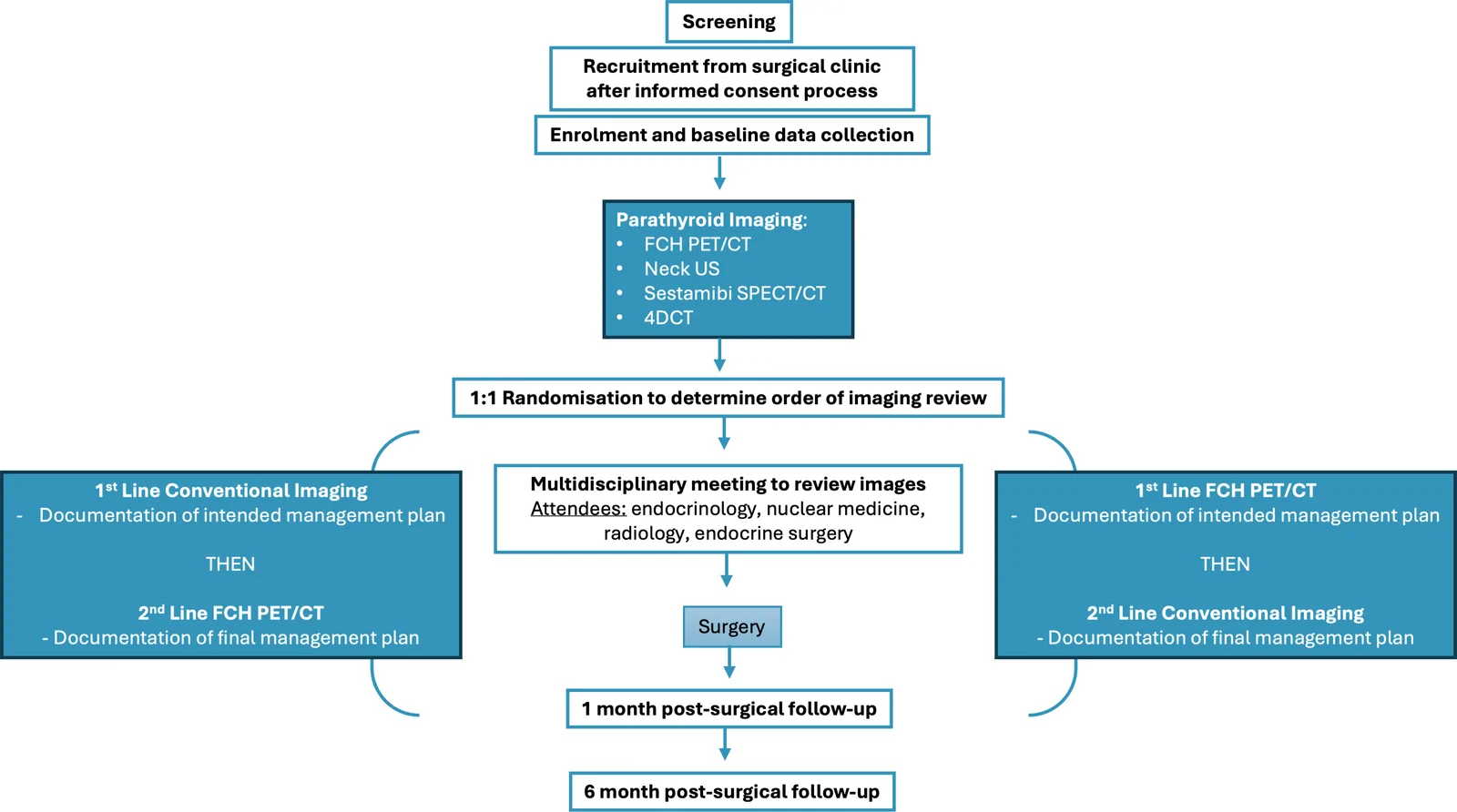
Trial schema. 4DCT, four-dimensional CT; FCH, [^18^F]fluorocholine; SPECT, single-photon emission computed tomography; US, ultrasound.

## Methods and analysis

### Study setting

We will enrol eligible patients referred for parathyroid surgery at one acute tertiary hospital site, the Royal Brisbane and Women’s Hospital (RBWH) in Brisbane, Australia.

### Eligibility criteria

Inclusion criteria:

1. Aged 18 or more years of age.
2. Biochemically confirmed PTH-dependent hypercalcaemia, defined as^11^
  - Serum calcium elevated above the reference range (eg, adjusted for serum albumin >2.6 mmol/L or ionised calcium concentration >1.32 mmol/L) with PTH in or above the reference range.
3. Criteria met for parathyroidectomy,^11 12^ confirmed by an endocrinologist.
  - Symptoms of hyperparathyroidism.
  - Asymptomatic hyperparathyroidism aged under 50 years.
  - Osteopenia, osteoporosis or significant bone loss over time (defined as a reduction greater than the least significant change defined by the International Society for Clinical Densitometry).
  - Raised urine calcium excretion (>250 mg/day in women; >300 mg/day in men^2^), renal calculus or deteriorating renal function (estimated glomerular filtration rate (eGFR) <60 mL/min/1.73 m^2^ in the absence of another explanation).
  - Calcium adjusted for serum albumin >2.75 mmol/L.
4. Able and willing to provide informed consent.

Exclusion criteria:

1. Estimated glomerular filtration rate below 30 ml/min/1.73 m^2^, performed within 3 months of screening or clinical suspicion for tertiary hyperparathyroidism, as determined by referring clinician or study investigator.
2. Current pregnancy or breastfeeding.
3. Clinical suspicion of parathyroid carcinoma, as determined by referring clinician or study investigator.
4. Use of colchicine, if unable to be withheld for 48 hours prior to FCH PET/CT, judged by referring clinician.
5. MEN1 syndrome, defined as either ^13^
  - Genetic: documented MEN1 gene mutation.
  - Familial: presence of one main MEN1 manifestation alongside first degree relative with MEN1 syndrome.
  - Clinical: presence of two main MEN1 manifestations.
6. Previous parathyroid surgery.
7. Proven or suspected familial hypocalciuric hypocalcaemia (FHH).
8. Documented severe allergy to intravenous iodinated contrast.

### Interventions

Participants will undergo both diagnostic imaging strategies (FCH PET/CT and conventional imaging with sestamibi SPECT/CT and 4DCT), unless previously performed within 12 months (table 1). All patients will receive a standard of care neck US to assist with operative planning, particularly to define the anatomic relationship between abnormal parathyroid tissue and the thyroid gland (ie, intra-thyroidal vs extra-thyroidal location).

**Table 1:** Timeline of study intervention and monitoring

	Screening period	Diagnostic imaging*	Multidisciplinary meeting	Surgery (within 12 weeks from screening)	1-month post-surgery follow-up	Second imaging review (3 months after initial review)	6-month post-surgery follow-up
Eligibility screen	x						
Written informed consent	x						
Allocation			x				
Documentation of intended management plan			x				
Blood test: calcium, albumin, PTH, vitamin D, creatinine	x				x		x
Bone mineral density and thoracolumbar X-ray (if not performed in previous 12 months)	x						
Urine 24-hour calcium excretion (if not performed in previous 12 months) and renal imaging (US or CT, if not performed in previous 5 years)	x						
bHCG in females of reproductive age if possibility of pregnancy		x					
Patient satisfaction measure					x		
Assessment of radiation and health service utilisation		x			x		
FCH PET/CT		x					
Neck US		x					
Sestamibi SPECT/CT		x					
4DCT		x					
Parathyroidectomy				x			
Interpretation of imaging			x			x	

* Imaging will not be repeated if performed within the previous 12 months from screening.

4DCT, four-dimensional CT; FCH, [18F]fluorocholine; PTH, parathyroid hormone; SPECT, single-photon emission tomography; US, ultrasound.

The neck US, sestamibi SPECT/CT and 4DCT will be interpreted as per standard care by a radiologist (neck US and 4DCT) or nuclear medicine specialist (sestamibi SPECT/CT). The FCH PET/CT will be performed at RBWH Department of Nuclear Medicine & PET Services and interpreted by a nuclear medicine specialist blinded to other imaging modalities.

All imaging studies for each participant will be reviewed at a monthly multidisciplinary team (MDT) meeting, attended by representatives from Endocrinology, Endocrine Surgery, Nuclear Medicine and Radiology. Participants will be randomised 1:1 to the order in which diagnostic strategy is reviewed first—either FCH PET/CT or conventional imaging (with sestamibi SPECT/CT then 4DCT). The neck US will be reviewed after both imaging strategies for final surgical planning and will be considered separate from each diagnostic strategy.

Participants’ imaging will be reviewed using study ID with clinicians blinded to patient name and radiologist/nuclear medicine specialists blinded to alternative imaging arm results. The study investigator will record the intended management plan at baseline and following first-line imaging and the final management plan after second-line imaging. The intended surgical approach, either MITP or BNE, will be documented according to a schema of positive, equivocal and negative results of each imaging technique. The management impact will be classified as

- High=change in management from BNE to MITP or confident localisation of an additional surgical target.
- Moderate=change in management approach but not straightforward MITP (eg, identified one or two possible foci to target during BNE) or improved confidence of localisation of an equivocal lesion identified on first-line imaging.
- Low=no change in management.

The findings of each modality, including neck US, will also be recorded individually.

Final clinical management of participants within this study will remain at the discretion of the individual endocrine surgeon working within a multidisciplinary framework. The reason for any deviation from the intended surgical approach will be recorded. As current best practice, it is expected that the majority of participants within the study will proceed to neck surgery within 12 weeks of randomisation. Participants will be followed up at 1 and 6 months after definitive surgery, with MDT re-discussion of operative findings, histology and postoperative biochemistry to confirm accurate localisation has been achieved. Consensus of single or multiglandular disease and gland location for comparison with imaging findings will be reached to determine accuracy. Any disagreements will be adjudicated by the MDT. Biochemical cure will be defined as normocalcaemia at 6 months post-surgery.^3^

#### FCH PET/CT

[^18^F]FCH has been validated at RBWH through an automated manufactured radiolabelling process. Quality control testing was aligned with British Pharmacopoeia standards for FCH and the processes implemented into the Q-TRaCE Pharmaceutical Quality System. [^18^F]FCH administration will be performed in accordance with EANM parathyroid imaging guideline recommendations.^8^ Images will be acquired using a Siemens Biograph 128 Vision 600 Edge-8R PET/CT scanner. Colchicine will be ceased for 48 hours prior to the scan where safe to do so. Nil other preparation will be required. Administered activity will be 1.5–2 MBq/kg, with a minimum activity of 100 MBq and a maximum activity of 200 MBq administered intravenously via an indwelling venous cannula, followed by a 40 mL normal saline flush. Early phase imaging will commence 5–10 min post-injection. If there are equivocal findings on the early FCH PET/CT, a delayed image may be requested by the reporting nuclear medicine specialist. Delayed images at 60–75 min post-injection will be acquired at 60 min.

Patients will be positioned supine, with early phase acquisition extending from the temporomandibular joints to the diaphragm. Delayed acquisition, when performed, will be reduced to the equivalent of one bed position, extending inferiorly from the temporomandibular joints. Continuous bed motion will be performed at 0.5 mm/s (equivalent 4 min/bed). PET images will be acquired with a matrix of 440×440 with a field of view of 780 mm. PET attenuation-corrected and non-attenuation-corrected images will be reconstructed using point spread function, time of flight (PSF+TOF) with five iterations and five subsets (5i5s), with a Gaussian filter of 3 mm. standardised uptake value (SUV) measurement method SUV-bw. The low-dose CT acquisition will be performed using a reference tube current of 80 mAs and a tube voltage of 120 kVp. The CT field of view will be 500 mm, with a pitch of 0.8 and collimation of 0.6 mm. Low-dose CT images will be reconstructed at two slice thicknesses: 0.75 mm and 2 mm. Images will be reconstructed using a 512×512 matrix and the Bf39f reconstruction kernel.

The FCH PET/CT will be reported as positive if a focal FCH avid lesion is identified in a typical location for a parathyroid adenoma correlating with a low-dose CT abnormality near the posterior thyroid, or ectopically along the embryonic descent pathway (fourth pouch superior glands near the tracheoesophageal groove and third pouch inferior glands more inferiorly within the thymus or mediastinum). Scans will be reported negative if no focal abnormal FCH uptake is identified, and equivocal if there is focal uptake in an atypical location or very faint uptake. Thyroid nodules and reactive lymph nodes will be reviewed in the context of other scan findings (including presence or absence of localised adenoma) and further investigations recommended if required. The location of uptake will be recorded (lateralisation to left/right, relationship to tracheal bisecting line (anterior=lower, posterior=upper) and thyroid, or ectopic position), intensity of uptake (SUVmax) and the maximum transverse CT diameter will be measured. Inter-observer reliability will be formally evaluated as a sub-study.

#### Conventional imaging

The sestamibi SPECT/CT and 4DCT will be performed according to the RBWH’s routine protocols. A minority of patients are anticipated to have already undergone equivalent conventional imaging within 12 months prior to surgical referral. These participants will not have imaging repeated as it would be unethical for research participants to receive unnecessary radiation exposure from repeating diagnostic quality imaging for a benign condition.

The sestamibi parathyroid imaging will consist of a dual-tracer subtraction technique and sestamibi SPECT/CT, using approximately 75Mbq of [^99m^Tc]pertechnetate for initial thyroid imaging and 700MBq of [^99m^Tc]sestamibi for parathyroid imaging. Pinhole imaging (right anterior oblique, RAO 30^o^, left anterior oblique, LAO 30^o^, anterior) of the neck will be performed after the injection of pertechnetate, to acquire each image for 4 min per view. Imaging will begin no earlier than 10 min after injection of [^99m^Tc]pertechnetate. Without moving the patient, [^99m^Tc]sestamibi will be injected, and pinhole images will be repeated after 10 min, to acquire each image for 4 min per view. A low-energy high-resolution collimator static image of the anterior mediastinum will be acquired for 4 min. A SPECT with low-dose CT will be performed of the neck and mediastinum after static images are complete. A dual-head gamma camera will perform non-circular rotation, acquiring 11 s per view, with number of views 60, degrees of rotation 180, matrix 256×256 with zoom 1.0. Delayed repeat imaging will be acquired 2 hours post-[^99m^Tc]sestamibi injection, comprising repeat planar and pinhole imaging of the neck and chest, with repeat SPECT and low-dose CT as indicated. Parathyroid subtraction will be performed per institution protocol. It is noted that not all imaging centres routinely perform [^99m^Tc]pertechnetate thyroid imaging for parathyroid subtraction imaging as part of their parathyroid scintigraphy studies. Images will be reconstructed and displayed, and sent to Picture Archiving and Communications Storage (PACS).

4DCT will be performed using a Philips IQon Spectral CT scanner. This will involve an initial non-contrast CT of the neck. Following this, an intravenous injection of 75 mL of non-ionic 350 or higher strength contrast, injected at 4 mL/s. Post-contrast imaging will then be performed using bolus tracking, including arterial phase at 10 s post threshold delay (typically 25–40 s after contrast injection) and during the venous phase (typically 60–80 s after contrast injection). The thin CT images will be sent to PACS along with 2 mm axial, coronal and sagittal multiplanar reformats for review. In the event of discordant results between sestamibi SPECT/CT and 4DCT, consensus will be reached after discussion within the MDT, with the latter weighted more heavily based on recognition of superior accuracy.

##### Modifications

If any clinical or imaging findings are suspicious for parathyroid carcinoma (eg, parathyroid lesion measuring greater than 3 cm and/or radiological evidence of invasion into surrounding structures) or MEN1, the participant will be discontinued from the trial and proceed to surgery as per standard of care for this condition.

##### Adherence

When gaining consent for participation in the trial, participants will be educated on the importance of attending the diagnostic imaging test required by the study protocol. If participants fail to attend appointments, they will be contacted via phone by a study investigator for a discussion of reasons and exploration of strategies to encourage adherence to the trial protocol.

##### Concomitant care

As this trial is assessing an imaging modality, concomitant medications are permitted (except colchicine, which is temporarily withheld for 48 hours prior to the FCH PET/CT) and will be recorded. A neck US, sestamibi SPECT/CT or 4DCT may be repeated at the surgeon’s discretion as per standard of care if the initial scans are not of sufficient quality to guide operative decisions.

### Outcomes

#### Primary outcome

1. The proportion of participants who had a change in intended management approach, from BNE to MITP, after first-line imaging strategy.

#### Secondary outcomes

1. In participants with biochemical cure after surgery, the proportion of each imaging test (FCH PET/CT, neck US, sestamibi SPECT/CT and 4DCT) to accurately localise parathyroid adenoma (ie, all clinically significant parathyroid adenomas have been localised).
2. In participants with negative or equivocal first-line imaging, the proportion who had a change in intended management approach after second-line imaging strategy.
3. The cost-effectiveness of first-line FCH PET/CT vs conventional imaging.
4. In participants with biochemical cure after imaging-guided MITP, the difference in total radiation exposure between using first-line conventional imaging compared with first-line FCH PET/CT (mSv per diagnostic strategy).
5. The environmental impact of each diagnostic strategy measured with life cycle assessment.
6. The patient/consumer experience of each diagnostic strategy.
7. The inter-observer agreement of FCH PET/CT, sestamibi SPECT/CT and 4DCT interpretation.

### Participant timeline

#### Sample size

The power calculation of this study is based on assessing whether FCH PET/CT is non-inferior to conventional imaging for the primary outcome of the proportion of participants whose intended management changed from BNE to MITP after first-line imaging. It is anticipated that for the conventional imaging strategy, 80% of patients will have a change in management. Assuming an actual difference between FCH PET/CT and conventional imaging outcome proportions of 0, a non-inferiority margin of −0.15, 126 patients (63 in each arm) achieves 80% power at a significance level of 0.025. The non-inferiority margin was determined in the context of wide variability in accuracy of conventional imaging techniques used in routine clinical practice.^5 6^ A 15% reduction in accuracy was judged to be appropriate by multidisciplinary consensus, based on the minimum clinically acceptable difference in management impact, given the additional benefits offered by FCH PET/CT. This margin was prespecified and set prior to commencing recruitment or review of any outcome data. Allowing for an additional 5% of potentially nonassessable patients, we plan to enrol a total of 134 participants. Calculations were carried out using a one-sided non-inferiority test of the difference between two proportions using PASS 2020 software.

#### Recruitment

All patients referred to the RBWH for surgical management of PTH-dependent hypercalcaemia will be screened against the inclusion/exclusion criteria by a study investigator. Screening all patients will minimise any potential bias in selection of participants.

#### Allocation

The random allocation sequence for 134 participants will be produced by an independent statistician using the blockrand package in R.^14^ Permuted random blocks of size 2, 4 and 6 will be used. This block randomisation ensures that the imbalance in group sizes does not exceed 3 at any point during recruitment. The block sizes will be randomly permuted to prevent study staff from knowing the size of the randomisation block at any point during recruitment, thus preserving allocation concealment.

##### Implementation

The day prior to each MDT, the allocation to one of two study arms (first-line imaging review with FCH PET/CT or conventional imaging) will be communicated to a study investigator. The allocation will not be communicated to the referring medical practitioner.

### Blinding

The nuclear medicine specialist reporting the FCH PET/CT will not have access to the patient’s medical record and will not be able to view prior imaging reports or images. Blinded interpretation will be performed by two nuclear medicine specialists, one at the time of the scan and one just prior to the MDT. In the event of disagreement, a third read will be undertaken by a different clinician.

As per standard care the neck US and 4DCT will be evaluated by a radiologist, and sestamibi SPECT/CT by a nuclear medicine specialist. The medical imaging specialist describing the conventional imaging findings will not have access to the FCH PET/CT images or report, as these are not available in the medical record or PACS until after the MDT.

Participants’ imaging will be reviewed at the MDT using study ID with clinicians blinded to patient name, and medical imaging specialists blinded to alternative imaging arm results. Accidental unblinding is unlikely to occur but if identified, would be recorded by the study monitor, disclosed in the results with participants excluded from the analysis. A second blind read of all exams will be performed 3 months after the last inclusion by a different clinician to estimate the inter-observer agreement.

### Data collection

Patient demographics will be recorded (age, sex, weight, height, address, ethnicity). Baseline clinical data performed as part of routine clinical care will be recorded including symptoms of PHPT, pathology (calcium, albumin, PTH, vitamin D, creatinine and eGFR within the previous 3 months and any genetic testing and 24-hour urine calcium excretion), prior and additional imaging (neck US, sestamibi SPECT/CT, 4DCT, bone mineral density, thoracolumbar X-ray, renal imaging with CT or US) and medical history. Reasons for drop-out, if occurring, will be recorded.

### Data management

Data will be collected via direct entry into the Research Electronic Data Capture (REDCap) software hosted by Queensland Health.^15^ This reduces ‘double handling’ of data. Numerical input fields will have range checks for data values to minimise the chance of data entry error. The data will be stored in the secure cloud-based Queensland Health REDCap server, with de-identified secure backup copies made weekly. Data will only be accessible by designated study investigators. Electronic and paper study files will be kept for 15 years at which point they will be securely destroyed.

### Health economic data collection and analysis

A trial-based cost-effectiveness will be undertaken according to international best practice methods in addition to a budget impact analysis to the hospital system.^16^ We will assess the costs, diagnostic outcomes and anticipated health benefits associated with FCH PET/CT compared with conventional imaging from a societal perspective. Healthcare resource use captured for all participants will be quantified, adjusted for inflation where necessary. Resources for the delivery of diagnostic imaging services will consider the radiotracers, imaging procedures, personnel and time, multidisciplinary meeting and all resources (tests, pathology) involved for the development and preparation of scanning services. Subsequent management of participants with surgery or other treatments will also be monitored and costed, as well as any adverse events. Patient travel time will be estimated from residential postcode and estimating travel time to the RBWH on Google Maps. Resources will be valued for costing purposes using publicly available sources, such as those published online via the Medicare Benefits Schedule (MBS) or national hospital costing schedules. Costs of the environmental impacts (radioactive waste, carbon footprint) will be included, where feasible according to newly developed methods.^17^ A Health Economics Analysis Plan will be developed, and the evaluation will conform to the Consolidated Health Economic Evaluation Reporting Standards (CHEERS) Checklist.^18^

Incremental cost-effectiveness ratios will be calculated. A cost-minimisation will be considered in the case of non-inferior outcomes. A resampling method using single imputation nested in bootstrapping will be used to assess uncertainty around the ratio values. CIs (95%) around ratios for the difference in mean costs and effects will be generated through the bootstrap percentile method using 1000 bootstrap replications. The findings will be interpreted according to acceptable thresholds in Australia for reimbursement of new technologies on the MBS, benchmarked against similar procedures within nuclear medicine.

### Patient satisfaction

Patient satisfaction with both diagnostic strategies will be assessed on a scale of 1–5 with an option for qualitative responses. Surveys will be completed electronically during attendance at the post-surgery follow-up appointment. The two modes of diagnostic imaging administration are anticipated to have different impacts on patient time and activity. To assess the impact on patient time and activity associated with complying with diagnostic imaging, the patient waiting time (assessed as the difference between time of the radiotracer administration and the time the scan commenced) and duration of the procedure (difference between the time the patient commences the scan and the time the scan is completed and patient leaves) will be assessed. Estimated patient travel from home to the imaging department will be included. Patient wait time in the department before commencing the scan will be excluded as this is multifactorial and mostly beyond the control of the study.

### Statistical methods

Analysis will be performed according to both per-protocol and intention-to-treat principles. Categorical variables will be described using frequency and percent, while continuous outcomes will be described using mean and SD, or median and IQR as appropriate. To assess whether FCH PET/CT is non-inferior to conventional imaging for the primary outcome of the proportion of participants who had a change in intended management approach after first-line imaging, the risk difference between groups will be calculated and accompanying CI derived using either the Newcombe and/or Farrington-Manning method as appropriate. The upper bound of the two-sided 95% CI for the conventional imaging minus the FCH PET/CT outcome measure will be compared with the pre-specified margin of error of 0.15. If the upper bound is less than the margin of error, non-inferiority will be established. If non-inferiority is achieved, superiority of the FCH PET/CT imaging group will thereafter be assessed.

Comparison of the proportion of each individual imaging test within a patient to accurately localise parathyroid adenoma in only patients with a biochemical cure after surgery will be undertaken using a binary logistic mixed model with patient as a random intercept or alternatively a generalised estimating equation clustered on patient. Along with imaging technique type, period and/or order of image read effects may be assessed in the model. A complementary analysis will be performed in all operated patients to ensure more complex cases are not excluded. Differences between groups for remaining categorical secondary outcomes will be compared using chi-square tests or Fisher’s exact tests for small cell counts. For continuous normally distributed outcomes, t-tests will be used or Mann-Whitney U tests for non-normally distributed outcomes. The statistical significance threshold will be set at an α<0.05.

If any incomplete data are identified, the participant would be excluded from the analysis and a sensitivity analysis performed with any missing data excluded. Incomplete histology is unlikely to occur as pathology is managed centrally and records will be complete. These data will be extracted from the hospital’s electronic medical record. Missing 6-month biochemical data are unlikely to occur as this is a routine part of medical care post parathyroidectomy. In the event that no 6-month blood collection takes place, the subsequent biochemistry result from 6 months until the conclusion of the study will be used (once biochemical cure has been achieved, it should be maintained unless in the rare event of metachronous multiglandular disease occurring in the subsequent 12-month period).

Inter-observer variability of FCH PET/CT, sestamibi SPECT/CT and 4DCT interpretation will be estimated using Cohen’s kappa coefficients between two independent observers for the first and second interpretations separately.

### Data monitoring

A Data Safety and Monitoring Board comprising relevant experts has been established, who will review trial data including cases of overcorrection and any serious adverse effects.

### Harms

Any acute adverse event experienced by the patient at the time of radiotracer administration will be recorded. Any toxicity will be graded by NCI Common Terminology Criteria for Adverse Events V.4.03 and its relationship to the examination will be described as unrelated, unlikely, possible, probable or definite. Any serious adverse events will be reported to the study sponsor and HREC.

FCH is similar to native physiologic choline, an essential nutrient which is incorporated into cell membrane synthesis of all mammalian cells.^19 20^ There are no known adverse effects related to the FCH itself. Potential drawbacks of FCH PET/CT include uptake of the radiotracer by thyroid nodules and inflammatory lymph nodes which may be a source of false-positive results.^19^

#### Radiation exposure

The radiation exposure from FCH PET/CT is typically lower than that of conventional parathyroid imaging modalities.^9 20^ Effective radiation doses will be reported in millisieverts (mSv). Overall radiation exposure from the two imaging approaches (FCH PET/CT vs conventional) will be tabulated and compared. Radiation exposure from FCH PET/CT and sestamibi SPECT/CT will be calculated for both the radiotracer administration and CT component. Radiation exposure endpoints will be assessed according to the individual radiation exposure received by trial participants determined from the radiopharmaceutical administered activity ([^18^F]FCH, [^99m^Tc]sestamibi or [^99m^Tc]) and CT dose-length product for each imaging modality.

#### Auditing

The trial will be subject to audits by the sponsor.

## Ethics and dissemination

### Research ethics approval

Ethics approval has been granted by the Metro North Human Research Ethics Committee (Project ID: 113012; HREC/2025/MNH/113012). Site-Specific Assessment approval was obtained prior to commencement of any study activities.

### Protocol amendments

If required, any protocol amendments will be sent to the study sponsor (Metro North Health) for review prior to submission to the approving Human Research Ethics Committees. Protocol modification will only be made with the approval of the study sponsor and investigation team and will be communicated to the Data Safety and Monitoring Board and all study personnel. Where necessary, participants will be notified and will sign an updated participant information and consent form.

### Consent

Participants will be contacted at their hospital appointment in person or via phone about potential study participation by a study team member. If they verbally consent to further discussion, the written participant information and consent form (see online supplemental file) will be sent via email or post, after which a study team member will discuss participation in more detail. Participants will be informed that their decision whether to participate in the study will not impact their access to routine care. Participants will be given the opportunity to read, discuss with support people and ask questions. Those willing to participate will sign the consent form.

### Confidentiality

Personal information will be collected only if consent to participate is obtained. These data will be stored on the secure cloud-based REDCap platform. Only authorised study personnel will be granted password-protected access. Only de-identified information will be published or reported.

### Access to data

De-identified data will be made available to The University of Queensland for the purposes of a higher degree and may be transferred to other organisations for the purposes of analysis. To maintain confidentiality, de-identified data will only be shared beyond the investigator group with permission of the principal investigator for purposes of statistical verification or pooled analysis when relevant for study or publication purposes.

### Ancillary and post-trial care

After completion of the trial period, patients will revert to usual care. The Endocrinology Unit is available at all hours via telephone to assist medical teams with the management of PHPT before, during or after the study.

### Dissemination policy

At the completion of the trial, all participants will be sent a letter summarising the study results and inviting them to contact the research team with any questions. The trial will be published in an international peer-reviewed journal. Abstracts will also be submitted to relevant national and international endocrine, endocrine surgery and nuclear medicine scientific meetings.

### Patient and public involvement statement

The trial was discussed with patients with PHPT during their appointment in the RBWH Endocrinology and Endocrine Surgery clinics. Comments were invited into the trial design, particularly around the requirement for all imaging modalities. Based on patient feedback, the outcome of patient satisfaction for each imaging modality, and in particular the duration of each test, was incorporated into the trial design as a secondary outcome.

## Supplementary material

10.1136/bmjopen-2026-122455 online supplemental file 1
