# Supplementary material for: Protocol for PARA-PET: a trial of [18F]fluorocholine PET/CT versus conventional imaging for primary hyperparathyroidism with randomised order of blinded imaging assessment
Source: BMJ Open. 2026 Oct 29;16(10):e122455. doi: 10.1136/bmjopen-2026-122455 (PMC13723825; doi:10.1136/bmjopen-2026-122455)
Supplement: Supplementary file 1 [file bmjopen-16-10-s001.pdf]

Adult providing own consent

Royal Brisbane & Women's Hospital

**Title**	PARA-PET: a randomised trial of [18F]fluorocholine PET/CT vs conventional imaging for primary hyperparathyroidism
**Short Title**	[18F]Fluorocholine PET for primary hyperparathyroidism
**HREC Number**	113012
**Protocol Number**	Version 3 – 17 February 2026
**Principal Investigator**	Dr Elizabeth Wootton<br>Endocrinologist, Royal Brisbane and Women's Hospital<br>PhD Candidate, University of Queensland
**Location**	Royal Brisbane and Women's Hospital
**Funding**	Queensland Technology Future Fund, RACP, NHMRC

Part 1 What does my participation involve?

1. Introduction

You are invited to take part in this research project as you have been referred for imaging to investigate your primary hyperparathyroidism at the Royal Brisbane and Women's Hospital (RBWH). This 'Participant Information Sheet/Consent Form' will tell you all about the clinical trial. Knowing what is involved will help you decide if you want to take part in the clinical trial.

Please read this information carefully. Ask questions about anything that you do not understand or want to know more about. Before deciding whether to take part, you might want to talk about it with a relative, friend or your doctor. Participation in this research is voluntary. If you do not wish to participate you are under no obligation to do so. You will receive the best possible care in relation to your treatment whether, or not, you participate.

If you decide you want to take part in the research project, you will be asked to sign the consent section.

By signing it you are telling us that you:

- Understand what you have read.
- Consent to take part in the research project.
- Consent to having an additional imaging scan, called a PET/CT scan with the [18F]FCH radiotracer.
- Consent to the use of your personal and health information as described.

You will be given a copy of this Participant Information and Consent Form to keep.

2. What is the purpose of this research?

Imaging scans are used in primary hyperparathyroidism to identify abnormal parathyroid glands before surgery. This makes sure the best type of surgery is performed. There are a number of different scans available. This study aims to compare the imaging scans we currently use (sestamibi and 4D-CT scans) to a newer scan called [^{18}F]Fluorocholine (FCH) PET/CT. This new scan is being assessed to see if it is better than the previous ones.

A PET/CT scan is a common type of imaging method. With the use of a substance called a radiotracer, it can show how parts of the body are functioning and is linked to a CT scan which shows the structure. The [18F]FCH radiotracer is used to highlight parathyroid glands that are overactive.

In this clinical trial, the research team would like to compare whether this new PET/CT scan with the [18F]FCH radiotracer performs as well as or better than our current imaging techniques. FCH PET/CT has already been shown to accurately detect abnormal parathyroid glands but has not yet been directly compared with 4D-CT.

3. What does participation in this research involve?

We aim to recruit 134 patients with primary hyperparathyroidism to participate in this study. This study commenced mid 2025 and will continue until we reach our target number of participants.

As part of your routine clinical care, you will be invited to attend the RBWH at appointed times to receive your standard imaging with sestamibi and 4D-CT. As part of this clinical trial, you will be required to attend the RBWH for the FCH PET/CT scan.. At your routine post surgery review, you will be requested to complete a survey about your experience with the imaging scans.
Please read this information sheet and consider if you are willing to participate. Prior to you having any scans you will meet with the research team to discuss your medical history and answer any questions about this trial. You will have three or more days to decide whether or not to participate. If you choose to participate, the team will ask you to sign this consent form.

The FCH PET/CT scan appointment will take approximately 2-3hrs at RBWH. You do not need to do any special preparation for the procedure, although we do recommend drinking an extra couple of glasses of water to make sure you are well hydrated.

Prior to the scan, you will have an injection of the 18F radiotracer through an intravenous cannula in a vein in your arm. The scan occurs 5-10 minutes after the injection. This is the “early” phase scan.

During the scan you will lie flat on your back, for approximately 15 minutes. After this scan, you will be seated in a comfortable recliner chair whilst your scan is reviewed by a doctor. If the doctor reviewing the “early” phase scan feels it is necessary you may receive a second scan between 60-75 minutes after the injection, otherwise you are able to leave. This will be a repeat of the first scan and is called the

"delayed" scan. At completion of scanning, you will have an opportunity to ask any more questions regarding the study.

If you decide to have surgery for your primary hyperparathyroidism, one and six months after surgery the research team will follow-up your progress via your medical records and may contact your treating doctor to gather information on your progress.

4. What do I have to do?

Please read through this information sheet and ask any questions you may have to the study team. If you are willing to participate in this clinical trial, please sign the consent form.

5. Do I have to take part in this research project?

Participation in any research project is voluntary. If you do not wish to take part you are under no obligation to do so. If you decide to take part and later change your mind you are free to withdraw from the project at any stage.
If you agree to participate and then withdraw, your images and clinical information obtained up to the time of your withdrawal will be included in the data analysis of this trial unless you stipulate otherwise. Your decision to take part, to not take part, or to withdraw consent after initially agreeing to take part, will not affect any future care or treatment you may require at the Royal Brisbane and Women's Hospital.

6. What are the alternatives to participation?

If you choose not to take part in this research project, you will receive routine care for primary hyperparathyroidism. This will involve receiving a sestamibi scan and 4D-CT scan prior to your surgery.

7. What are the possible benefits of taking part?

We cannot guarantee or promise that you will receive any personal benefits from this research. However the FCH PET/CT scan may be more accurate in identifying the abnormal parathyroid gland(s) causing your primary hyperparathyroidism and may make surgery quicker and safer to perform. We hope that the knowledge gained in this research will result in the new scan becoming more widely available which would benefit other patients with primary hyperparathyroidism in the future.

8. What are the possible risks and disadvantages of taking part?

This research study involves exposure to a small amount of extra radiation. As part of everyday living, everyone is exposed to naturally occurring background radiation and receive a dose of about 2 millisieverts (mSv) each year. The effective dose from this additional study is about 12 mSv, which is similar to that received from many other imaging scans. At this level, no harmful effects of radiation have been shown as any effect is too small to measure. For any imaging test involving radiation you should notify study staff if you may be pregnant. If you are pregnant you would not be eligible to participate in this study.

There is also a small risk associated with any injection such as bruising and swelling of the injection site or very rarely infection or a blood clot. An anaphylactic reaction (severe allergy) to the radiotracer has never been seen before but is theoretically possible and may result in symptoms such as difficulty breathing, racing heart and swelling of the lips. Some patients experience claustrophobia when lying in the scanner. You will be required to attend an additional appointment at the RBWH to receive your FCH PET/CT scan. You will be reimbursed for your parking expenses to attend this extra scan appointment.

9. What if new information arises during this research project?

The results of this study will not be available until after the end of the project. They will be published in an anonymised way in a peer reviewed medical journal. This new information will be used to improve the care of future patients with primary hyperparathyroidism.

10. Can I have other treatments during this research project?

Being involved in this research will not limit your option for other tests that will benefit your care for primary hyperparathyroidism.

11. What if I withdraw from this research project?

Please notify a member of the research team if you decide to withdraw from the study. There are no health risks associated with withdrawing from this study. If you withdraw from this study you are allowed to withdraw consent for your data to be used in the analysis.

12. Could this research project be stopped unexpectedly?

It is unlikely that this will occur and will not have any impact on your care as the research only involves receiving an extra scan in a research capacity.

13. What happens when the research project ends?

We intend to publish the results in an anonymised fashion in a peer-reviewed medical journal. If you would like to receive a copy of the results please indicate this on the consent form.

Part 2
How is the research project being conducted?

14. What will happen to information about me?

To maintain your privacy, data collected by your doctor and hospital staff is only provided to ethics committee representatives and study organisers with a code number rather than details that could identify you. The data will be kept on a file in a computer which is password protected. The computer is secured in a locked facility. Only the researcher/ investigator responsible for data collection will have unlimited access to this information.

By signing the consent form you consent to the study doctor and relevant research staff collecting and using personal information about you for the research project. This may include all test results and medical history pertaining to your primary hyperparathyroidism that has been gathered before signing this consent as part of your routine medical care. Any information obtained in connection with this research project that can identify you will remain confidential.

Your information will be used for the purpose of this research project and will only be disclosed with your permission, except as required by law. De-identified data will be made available through the University of Queensland for analysis as part of a research higher degree for one of the study team. It may also be shared with other researchers in accordance with Metro North Health Human Research Ethics Committee approval.

It is anticipated that the results of this research project will be published and/or presented in a variety of forums. In any publication and/or presentation, information will be provided in such a way that you cannot be identified, except with your permission. Your anonymised images may be used for future research or educational activities, including within other national projects. Personal details i.e. name, date of birth will not be published.

Information about you may be obtained from your health records held at this and other health services for the purpose of this research. By signing the consent form you agree to the study team accessing health records if they are relevant to your participation in this research project. Information about your participation in this research project will be recorded in your health records.

In accordance with relevant Australian and Queensland privacy and other relevant laws, you have the right to request access to your information collected and stored by the research team. You also have the right to request that any information with which you disagree be corrected. Please contact the study team member named at the end of this document if you would like to access your information.

Any information obtained for the purpose of this research project that can identify you will be treated as confidential and securely stored. It will be disclosed only with your permission, or as required by law. Please note the data derived from the study will be retained for 15 years according to the regulatory requirement for clinical trial.

15. Complaints and compensation

If you suffer any injuries or complications because of this research project you should contact the research team as soon as possible and you will be assisted with arranging appropriate medical treatment. If you are eligible for Medicare you can receive the necessary care required to treat the injury or complication, free of charge, as a public patient in any Australian public hospital.

16. Who is organising and funding the research?

This research is being conducted by the Nuclear Medicine Department of the Royal Brisbane and Women's Hospital. Funding has been received from the Queensland Technology Future Fund and the

Royal Australasian College of Physicians (RACP). No member of the research team will receive a personal financial benefit from your involvement in this research project (other than their ordinary wages). One of the study team will write the findings from this research in their PhD thesis for submission to The University of Queensland. They have received funding from the National Health and Medical Research Council (NHMRC).

17. Who has reviewed the research project?

This project will be carried out according to the *National Statement on Ethical Conduct in Human Research (2023)*. This statement has been developed to protect the interests of people who agree to participate in human research studies. This study has been reviewed and approved by the Metro North Human Research Ethics Committee. Should you wish to discuss the study in relation to your rights as a participant, or should you wish to make an independent complaint, you may contact the Coordinator or chairperson, Metro North Human Research Ethics Committee, Herston, Qld, 4029 or telephone (07) 3646 5280, or e-mail: MetroNorthResearch-Ethics@health.qld.gov.au

18. Further information and who to contact

The person you may need to contact will depend on the nature of your query.

If you want any further information concerning this project or if you have any medical problems which may be related to your involvement in the project (for example, any side effects), you can contact the principal study doctor via main switch (07) 3646 8111 or any of the following people:

Clinical contact person

Name	Dr Elizabeth Wootton
Position	Principal Investigator
Telephone	(07) 3646 8111 and ask to be contacted
Email	Elizabeth.wootton@heath.qld.gov.au

For matters relating to research at the site at which you are participating, the details of the local site complaints person are:

Complaints contact person

Name	Metro North Office of Research
Position	Research Governance Officer
Telephone	(07) 3647 9550
Email	MetroNorthResearch-RGO@health.qld.gov.au

If you have any complaints about any aspect of the project, the way it is being conducted or any questions about being a research participant in general, then you may contact:

Reviewing HREC approving this research and HREC Executive Officer details

Reviewing HREC name	Metro North Health HREC
Position	HREC Cordinator
Telephone	07 3646 5280
Email	MetroNorthResearch-Ethics@health.qld.gov.au

Consent Form - *Adult providing own consent*

**Title**	PARA-PET: a randomised trial of [18F]fluorocholine PET/CT vs conventional imaging for primary hyperparathyroidism
**Short Title**	[18F]fluorocholine PET for primary hyperparathyroidism
**HREC Number**	113012
**Protocol Number**	Version 3 – 17 February 2026
**Principal Investigator**	Dr Elizabeth Wootton
**Location**	Royal Brisbane and Women's Hospital

Declaration by Participant

I have read the Participant Information Sheet or someone has read it to me in a language that I understand.
I have had an opportunity to ask questions and I am satisfied with the answers I have received.
The nature, purpose and risks of the research project have been explained to me. I understand them and agree to take part.
I freely agree to participate in this research project as described and understand that I am free to withdraw at any time during the study without affecting my future health care.
I understand that if I participate in and then withdraw from the study, that any data collected to that point of withdrawal for the purposes of this study, will be retained for data analysis.
I understand that data arising from this study may be used for future research
I understand that I will be given a signed copy of this document to keep.
I understand that authorised representatives of the Sponsor, regulatory authorities and ethics committee representatives may be granted direct access to my medical records.

Name of Participant (please print) ____________________

Signature____________________Date__________

Declaration by Study Doctor/Senior Researcher†

I have given a verbal explanation of the research project, its procedures and risks, and the implications of withdrawal from the research project and I believe that the participant has understood that explanation.

Name of Study Doctor/
Senior Researcher† (please print) ____________________

Signature____________________Date__________

† A senior member of the research team must provide the explanation of, and information concerning, the research project.

Note: All parties signing the consent section must date their own signature.

Withdrawal of Consent Form

**Title**	PARA-PET: a randomised trial of [18F]fluorocholine PET/CT vs conventional imaging for primary hyperparathyroidism
**Short Title**	[18F]fluorocholine PET for primary hyperparathyroidism
**HREC Number**	113012
**Protocol Number**	Version 3 – 17 February 2026
**Principal Investigator**	Dr Elizabeth Wootton
**Location**	Royal Brisbane and Women's Hospital

Declaration by Participant

I wish to withdraw from participation in the above research project and understand that such withdrawal will not affect my routine treatment, my relationship with those treating me or my relationship with The Royal Brisbane and Women's Hospital.

I understand that any data collected up to the point of my withdrawal for the purposes of this study, will be retained for data analysis.

Name of Participant (please print) ______________________________

Signature ______________________ Date ______________________

Reason for withdrawal (optional):

Declaration by Study Doctor/Senior Researcher†

I have given a verbal explanation of the implications of withdrawal from the research project and I believe that the participant has understood that explanation.

Name of Study Doctor/
Senior Researcher† (please print) ______________________________

Signature ______________________ Date ______________________

† A senior member of the research team must provide the explanation of and information concerning withdrawal from the research project.

Note: All parties signing the consent section must date their own signature.
