# Supplementary material for: Protocol for PARA-PET: a trial of [18F]fluorocholine PET/CT versus conventional imaging for primary hyperparathyroidism with randomised order of blinded imaging assessment
Source: BMJ Open. 2026 Oct 29;16(10):e122455. doi: 10.1136/bmjopen-2026-122455 (PMC13723825; doi:10.1136/bmjopen-2026-122455)
Supplement: Supplementary file 2 [file bmjopen-2026-122455.reviewer_comments.pdf]

UNOFFICIAL

PEER REVIEW HISTORY

BMJ Open publishes all reviews undertaken for accepted manuscripts. Reviewers are asked to complete a checklist review form and are provided with free text boxes to elaborate on their assessment. These free text comments are reproduced below.

ARTICLE DETAILS

Title (Provisional)

A protocol for PARA-PET: a trial of [18F]fluorocholine PET/CT vs conventional imaging for primary hyperparathyroidism with randomised order of blinded imaging assessment

Authors

Wootton, Elizabeth; Wong, Clement; Donovan, Peter; Mcleod, Donald S. A.; Grossmann , M; Kyle, Samuel; Campbell, Louise; Pelecanos, Anita; Collins, Louisa G; Francis, Roslyn; Pattison, David A

VERSION 1 - REVIEW

**Reviewer**	**1**
**Name**	**Gong, Qiyong**
**Affiliation**	**West China Xiamen Hospital of Sichuan University**
**Date**	**25-Jul-2026**
**COI**	**None**

This protocol addresses an important clinical question: whether [18F]fluorocholine (FCH) PET/CT has a management impact non-inferior to that of the current multimodality work-up for primary hyperparathyroidism, and whether it offers advantages in examination time, radiation exposure, cost-effectiveness, and environmental impact. The prospective design, randomised order of image review, and multidisciplinary decision-making process are strengths. However, the manuscript requires substantial clarification regarding the object of randomisation, preservation of blinding, non-inferiority framework, and reproducibility of the imaging protocols. The following points should be addressed before the protocol can be considered sufficiently clear and reproducible.

1. The study design should be described more precisely. Participants are not randomised to receive FCH PET/CT or conventional imaging; all undergo both strategies, and randomisation determines the order of blinded review. The title, abstract, and Methods should make this clear.

2. The blinding procedure needs fuller explanation. Some participants may have prior imaging reports in the medical record. Please state how these results are concealed from the MDT, and how accidental unblinding will be documented and analysed.

UNOFFICIAL

3. The 15% non-inferiority margin needs clinical justification. Please explain why this degree of loss in management impact would remain acceptable, confirm that the margin was prespecified, and state when it was set in relation to recruitment and any review of outcome data.

4. Restricting the accuracy analysis to patients with biochemical cure may exclude important localisation failures. The planned analysis may be retained, but a complementary analysis in all operated patients should be prespecified. The handling of failed surgery, incomplete histology, and missing six-month biochemical data should be stated.

5. Please explain how operative findings, histopathology, gland location, postoperative calcium/PTH, and six-month cure are combined, and how disagreements are adjudicated.

6. The imaging protocols need enough technical detail to be reproducible. For FCH PET/CT, please report scanner model, acquisition and reconstruction parameters, CT protocol, SUV measurement method, and criteria for delayed imaging. Comparable details are needed for sestamibi SPECT/CT and 4DCT; a supplementary table would be suitable.

7. The definitions of positive, negative, and equivocal FCH PET/CT are too broad. In particular, 'faint uptake' is subjective. Please provide a structured reading scheme that incorporates CT appearance and common false-positive findings, including thyroid nodules and inflammatory lymph nodes. Please also state how equivocal findings will be handled in the accuracy analysis.

8. Radiation exposure should be reported separately for the radiopharmaceutical and CT components of FCH PET/CT and sestamibi SPECT/CT, together with the dose from 4DCT. The manuscript should distinguish the radiation actually received by trial participants from the estimated dose associated with each first-line strategy. The stated approximate effective dose of 12 mSv for FCH PET/CT should be linked to the actual CT protocol.

9. Please define the two imaging strategies consistently. It is not clear whether neck ultrasound is part of both strategies, part of conventional imaging only, or available only for final surgical planning. This should be stated at each stage of the MDT review.

VERSION 1 - AUTHOR RESPONSE

Reviewer: 1

Dr. Qiyong Gong, West China Xiamen Hospital of Sichuan University

Comments to the Author:

This protocol addresses an important clinical question: whether [18F]fluorocholine (FCH) PET/CT has a management impact non-inferior to that of the current multimodality work-up for primary hyperparathyroidism, and whether it offers advantages in examination time, radiation exposure, cost-effectiveness, and environmental impact. The prospective design, randomised order of image review, and multidisciplinary decision-making process are strengths. However, the manuscript requires substantial clarification regarding the object of randomisation, preservation of blinding, non-inferiority framework, and reproducibility of the imaging protocols. The following points should be addressed before the protocol can be considered sufficiently clear and reproducible.

1. The study design should be described more precisely. Participants are not randomised to receive FCH PET/CT or conventional imaging; all undergo both strategies, and randomisation determines the order of blinded review. The title, abstract, and Methods should make this clear.

- We have amended the title to:
  - A protocol for PARA-PET: a trial of [18F]fluorocholine PET/CT vs conventional imaging for primary hyperparathyroidism with randomised order of blinded imaging assessment
- We have amended the Abstract, line 80:
  - This study will be the first prospective trial to assess the management impact of FCH PET/CT compared to sestamibi SPECT/CT and 4DCT in individuals with primary hyperparathyroidism, with randomised order of blinded imaging review
- We have amended the Trial Design section, line 166:
  - We will conduct a single-centre prospective non-inferiority trial of FCH PET/CT compared to conventional imaging in patients requiring surgical management of PTH-dependent hypercalcaemia (Figure 1). All participants will receive both imaging strategies with randomisation determining the order of blinded imaging review

2. The blinding procedure needs fuller explanation. Some participants may have prior imaging reports in the medical record. Please state how these results are concealed from the MDT, and how accidental unblinding will be documented and analysed.

- We have amended the Blinding section, line 405:
  - The nuclear medicine specialist reporting the FCH PET/CT will not have access to the patient's medical record and will not be able to view prior imaging reports or images. Blinded interpretation will be performed by two nuclear medicine specialists, one at the time of the scan and one just prior to the MDT. In the event of disagreement, a third read will be undertaken by a diberent clinician.
  - As per standard care the neck US and 4DCT will be evaluated by a radiologist, and sestamibi SPECT/CT by a nuclear medicine specialist. The medical imaging specialist describing the conventional imaging findings will not have access to the FCH PET/CT images or report, as these are not available in the medical record or PACS until after the MDT.
  - Participants' imaging will be reviewed at the MDT using study ID with clinicians blinded to patient name, and medical imaging specialists blinded to alternative imaging arm results. Accidental unblinding is unlikely to occur but if identified, would be recorded by the study monitor, disclosed in the results with participants excluded from the analysis. A second blind read of all exams will be performed three months after the last inclusion by a diberent clinician to estimate the inter-observer agreement.

3. The 15% non-inferiority margin needs clinical justification. Please explain why this degree of loss in management impact would remain acceptable, confirm that the margin was prespecified, and state when it was set in relation to recruitment and any review of outcome data.

- We have added the following statement to the "Sample size" section, line 378:
  - "The non-inferiority margin was determined in the context of wide variability in accuracy of conventional imaging techniques used in routine clinical practice [5,6]. A 15% reduction in accuracy was judged to be appropriate, given the additional benefits offered by FCH PET/CT. This margin was pre-specified and set prior to commencing recruitment or review of any outcome data."

4. Restricting the accuracy analysis to patients with biochemical cure may exclude important localisation failures. The planned analysis may be retained, but a complementary analysis in all operated patients should be prespecified. The handling of failed surgery, incomplete histology, and missing six-month biochemical data should be stated.

- Thank you for this excellent observation. We have added the following statement to

Statistical Methods section, line 502:

- A complementary analysis will be performed in all operated patients to ensure more complex cases are not excluded.

- We have also added an additional paragraph to line 510:
  - If any incomplete data is identified, the participant would be excluded from the analysis and a sensitivity analysis performed with any missing data excluded. Incomplete histology is unlikely to occur as pathology is managed centrally and records will be complete. This data will be extracted from the hospital's electronic medical record. Missing six-month biochemical data is unlikely to occur as this is a routine part of medical care post parathyroidectomy. In the event that no six-month blood collection takes place, the subsequent biochemistry result from six months until the conclusion of the study will be used (once biochemical cure has been achieved, it should be maintained unless in the rare event of metachronous multiglandular disease occurring in the subsequent 12-month period).

5. Please explain how operative findings, histopathology, gland location, postoperative calcium/PTH, and six-month cure are combined, and how disagreements are adjudicated.

- Operative findings and histopathology are obtained from the electronic medical record, and post-operative calcium and six-month calcium results are obtained from pathology laboratory database. As per line 256, biochemical cure is defined as normocalcaemia at six-months post-operation and by definition indicates correct surgical localisation and excision of abnormal parathyroid glands.
- We have added the following statement to the Intervention section, line 254:
  - Consensus of single or multiglandular disease and gland location for comparison with imaging findings will be reached to determine accuracy. Any disagreements will be adjudicated by the MDT.

6. The imaging protocols need enough technical detail to be reproducible. For FCH PET/CT, please report scanner model, acquisition and reconstruction parameters, CT protocol, SUV measurement method, and criteria for delayed imaging. Comparable details are needed for sestamibi SPECT/CT and 4DCT; a supplementary table would be suitable.

- We have added the following detail to enhance reproducibility to the Interventions section:
  - Images will be acquired using a Siemens Biograph 128 Vision 600 Edge-8R PET/CT scanner. Colchicine will be ceased for 48 hours prior to the scan where safe to do so. Nil other preparation is required. Administered activity will be 1.5–2 MBq/kg, with a minimum activity of 100 MBq and a maximum activity of 200 MBq, administered intravenously via an indwelling venous cannula, followed by a 40 mL normal saline flush. Early phase imaging will commence 5–10 minutes post-injection. Delayed images at 60–75 minutes post-injection will be acquired at 60 minutes if early images do not confidently localise a parathyroid adenoma(s).
  - Patients will be positioned supine, with early phase acquisition extending from the temporomandibular joints to the diaphragm. Delayed acquisition, when performed, will be reduced to the equivalent of one bed position, extending inferiorly from the temporomandibular joints. Continuous bed motion will be performed at 0.5 mm/sec (equivalent 4 min/bed). PET images will be acquired with matrix of 440 × 440 with a field of view (FOV) of 780 mm. PET attenuation-corrected (AC) and non-attenuation-corrected (NAC) images will be reconstructed using PSF + TOF with five iterations and five subsets (5i5s), with a Gaussian filter of 3 mm. SUV measurement method SUV-bw. The low-dose CT acquisition will be performed using a reference tube current of 80 mAs and a tube voltage of 120 kVp. The CT field of view will be 500 mm, with a pitch of 0.8

and collimation of 0.6 mm. Low-dose CT images will be reconstructed at two slice thicknesses: 0.75 mm and 2 mm. Images will be reconstructed using a 512 × 512 matrix and the Bf39f reconstruction kernel.

- The sestamibi SPECT/CT will be performed as per the imaging centre's routine protocol. The majority of scans will occur at the RBWH where this will consist of a dual-tracer approach, using approximately 75Mbq of [^{99m}Tc] (pertechnetate) for initial thyroid imaging and 750MBq of [^{99m}Tc]Sestamibi for parathyroid imaging. Pinhole imaging of the neck will be performed after the injection of pertechnetate. Planar and pinhole imaging will then be performed after the injection of sestamibi, including the neck and chest. SPECT with low-dose CT will be performed of the neck and mediastinum. Following this delayed post-sestamibi planar and pinhole imaging will be performed of the neck and chest. It is noted that not all imaging centres routinely perform the pertechnetate thyroid imaging as part of their parathyroid scintigraphy studies.
- The 4DCT will also be performed as per the imaging centre's routine protocols. Similarly, the majority of scans will occur at the RBWH and will be performed using a Philips IQon Spectral CT scanner. This will involve an initial non-contrast CT of the neck. Following this, an intravenous injection of 75mL of non-ionic 350 or higher strength contrast, injected at 4mls/sec. Post-contrast imaging will then be performed using bolus tracking, including arterial phase at 10 seconds post threshold delay (typically 25-40 seconds after contrast injection) and during the venous phase (typically 60-80 seconds after contrast injection). The thin CT images will be sent to Picture Archiving and Communications Storage (PACS) along with 2 mm axial, coronal and sagittal multiplanar reformats (MPRs) for review.

7. The definitions of positive, negative, and equivocal FCH PET/CT are too broad. In particular, 'faint uptake' is subjective. Please provide a structured reading scheme that incorporates CT appearance and common false-positive findings, including thyroid nodules and inflammatory lymph nodes. Please also state how equivocal findings will be handled in the accuracy analysis.

- We have added the following detail to the FCH PET/CT section, line 287:
  - The FCH PET/CT will be reported as positive if a focal FCH avid lesion is identified in a typical location for a parathyroid adenoma correlating with a low-dose CT abnormality near the posterior thyroid, or ectopically along the embryonic descent pathway (4th pouch superior glands near the tracheoesophageal groove and 3rd pouch inferior glands more inferiorly within the thymus or mediastinum). Scans will be reported negative if no focal abnormal FCH uptake is identified, and equivocal if there is focal uptake in an atypical location or very faint uptake. Thyroid nodules and reactive lymph nodes will be reviewed in the context of other scan findings (including presence or absence of localised adenoma) and further investigations recommended if required. The location of uptake will be recorded (lateralisation, left / right, relationship to thyroid, upper / lower, or ectopic position), intensity of uptake (SUVmax) and when possible, the maximum transverse CT diameter will be measured.

8. Radiation exposure should be reported separately for the radiopharmaceutical and CT components of FCH PET/CT and sestamibi SPECT/CT, together with the dose from 4DCT. The manuscript should distinguish the radiation actually received by trial participants from the estimated dose associated with each first-line strategy. The stated approximate effective dose of 12 mSv for FCH PET/CT should be linked to the actual CT protocol.

- We have updated the Radiation Exposure section, line 541:
  - The radiation exposure from FCH PET/CT is typically lower than that of conventional parathyroid imaging modalities [9, 20]. Ebective radiation doses will

be reported in millisieverts (mSv). Overall radiation exposure from the two imaging approaches (FCH PET/CT vs conventional) will be tabulated and compared. Radiation exposure from FCH PET/CT and sestamibi SPECT/CT will be calculated for both the radiotracer administration and CT component.

Radiation exposure endpoints will be assessed according to the individual radiation exposure received by trial participants determined from the radiopharmaceutical administered activity ([^{18}F]FCH, [^{99m}Tc]Sestamibi or [^{99m}Tc]) and CT dose length product (DLP) for each imaging modality.

9. Please define the two imaging strategies consistently. It is not clear whether neck ultrasound is part of both strategies, part of conventional imaging only, or available only for final surgical planning. This should be stated at each stage of the MDT review.

- We apologise that this is unclear. We have amended the "Interventions" section, line 228 and 246:
    - Participants will be randomised 1:1 to the order in which diagnostic strategy is reviewed first – either FCH PET/CT or conventional imaging (with sestamibi SPECT/CT then 4DCT). The neck US will be reviewed after both imaging strategies for final surgical planning and will be considered separate from each diagnostic strategy.
    - The findings of each modality, including neck US, will also be recorded individually.
- The abstract has also been updated to, line 58:
    - 134 patients with primary hyperparathyroidism referred for surgery will receive FCH PET/CT in addition to conventional imaging (sestamibi SPECT/CT and 4DCT), as well as neck US for operative planning.

VERSION 2 - REVIEW

**Reviewer**	**1**
**Name**	**Gong, Qiyong**
**Affiliation**	**West China Xiamen Hospital of Sichuan University**
**Date**	**16-Sep-2026**
**COI**	

---

1. Please clarify whether FCH PET/CT interpretation will incorporate additional standardised criteria, including visual scoring systems, semi-quantitative parameters (e.g., SUV measurements), anatomical correlation on CT, and reader confidence assessment, to improve reproducibility and reduce inter-reader variability.

2. Regarding the non-inferiority margin, the authors should clarify the clinical rationale for selecting the 15% margin. The authors are encouraged to state that this margin was selected based on multidisciplinary consensus regarding the minimum clinically acceptable difference in management impact.

3. The revised manuscript provides improved descriptions of the FCH PET/CT protocol; however, technical details for conventional imaging modalities remain insufficient. To ensure methodological reproducibility and enable a fair comparison between imaging strategies, the authors should provide additional information regarding the acquisition protocols for

UNOFFICIAL

sestamibi SPECT/CT and 4DCT, including relevant imaging parameters and reconstruction methods, where applicable.

VERSION 2 - AUTHOR RESPONSE

Reviewer: 1
Dr. Qiyong Gong, West China Xiamen Hospital of Sichuan University

1. Please clarify whether FCH PET/CT interpretation will incorporate additional standardised criteria, including visual scoring systems, semi-quantitative parameters (e.g., SUV measurements), anatomical correlation on CT, and reader confidence assessment, to improve reproducibility and reduce inter-reader variability.

- Yes, we can clarify that FCH is reported according to the following standardised criteria, with semiquantitative parameters and anatomical correlation on low-dose CT. Formal evaluation of interobserver reliability will also be performed. We have added the following detail to line 277:
    - The FCH PET/CT will be reported as positive if a focal FCH avid lesion is identified in a typical location for a parathyroid adenoma correlating with a low-dose CT abnormality near the posterior thyroid, or ectopically along the embryonic descent pathway (4th pouch superior glands near the tracheoesophageal groove and 3rd pouch inferior glands more inferiorly within the thymus or mediastinum). Scans will be reported negative if no focal abnormal FCH uptake is identified, and equivocal if there is focal uptake in an atypical location or very faint uptake. Thyroid nodules and reactive lymph nodes will be reviewed in the context of other scan findings (including presence or absence of localised adenoma) and further investigations recommended if required. The location of uptake will be recorded (lateralisation to left / right, relationship to tracheal bisecting line [anterior = lower, posterior = upper] and thyroid, or ectopic position), intensity of uptake (SUVmax) and the maximum transverse CT diameter will be measured. Inter-observer reliability will be formally evaluated as a sub-study.

2. Regarding the non-inferiority margin, the authors should clarify the clinical rationale for selecting the 15% margin. The authors are encouraged to state that this margin was selected based on multidisciplinary consensus regarding the minimum clinically acceptable diYerence in management impact.

- We have added the following detail to line 384:
    - The non-inferiority margin was determined in the context of wide variability in accuracy of conventional imaging techniques used in routine clinical practice [5, 6]. A 15% reduction in accuracy was judged to be appropriate by multidisciplinary consensus, based on the minimum clinically acceptable difference in management impact, given the additional benefits offered by FCH PET/CT.

3. The revised manuscript provides improved descriptions of the FCH PET/CT protocol; however, technical details for conventional imaging modalities remain insuYicient. To ensure methodological reproducibility and enable a fair comparison between imaging strategies, the authors should provide additional information regarding the acquisition protocols for sestamibi SPECT/CT and 4DCT, including relevant imaging parameters and reconstruction methods, where applicable.

- We have added the following detail to line 291:
    - The sestamibi SPECT/CT and 4DCT will be performed according to the RBWH's routine protocols. A minority of patients are anticipated to have already undergone equivalent conventional imaging within 12 months prior to surgical referral. These participants will not have imaging repeated as it would

UNOFFICIAL

be unethical for research participants to receive unnecessary radiation exposure from repeating diagnostic quality imaging for a benign condition.

- The sestamibi parathyroid imaging will consist of a dual-tracer subtraction technique and sestamibi SPECT/CT, using approximately 75Mbq of [^{99m}Tc] pertechnetate for initial thyroid imaging and 700MBq of [^{99m}Tc]Sestamibi for parathyroid imaging. Pinhole imaging (RAO 30°, LAO 30°, Anterior) of the neck will be performed after the injection of pertechnetate, to acquire each image for four minutes per view. Imaging will begin no earlier than ten minutes after injection of [^{99m}Tc]pertechnetate**.** Without moving the patient, [^{99m}Tc]Sestamibi will be injected, and pinhole images will be repeated after ten minutes, to acquire each image for four minutes per view. A low-energy high resolution (LEHR) collimator static image of the anterior mediastinum will be acquired for four minutes. A SPECT with low-dose CT will be performed of the neck and mediastinum after static images are complete. A dual head gamma camera will perform non-circular rotation, acquiring 11 seconds per view, with number of views 60, degrees of rotation 180, matrix 256x256 with zoom 1.0. Delayed repeat imaging will be acquired two hours post-[^{99m}Tc]Sestamibi injection, comprising repeat planar and pinhole imaging of the neck and chest, with repeat SPECT and low-dose CT as indicated. Parathyroid subtraction will be performed per institution protocol. It is noted that not all imaging centres routinely perform [^{99m}Tc]pertechnetate thyroid imaging for parathyroid subtraction imaging as part of their parathyroid scintigraphy studies. Images will be reconstructed and displayed, and sent to Picture Archiving and Communications Storage (PACS).
- The 4DCT will be performed using a Philips IQon Spectral CT scanner. This will involve an initial non-contrast CT of the neck. Following this, an intravenous injection of 75mL of non-ionic 350 or higher strength contrast, injected at 4mls/sec. Post-contrast imaging will then be performed using bolus tracking, including arterial phase at 10 seconds post threshold delay (typically 25-40 seconds after contrast injection) and during the venous phase (typically 60-80 seconds after contrast injection). The thin CT images will be sent to PACS along with 2 mm axial, coronal and sagittal multiplanar reformats (MPRs) for review. In the event of discordant results between sestamibi SPECT/CT and 4DCT, consensus will be reached after discussion within the MDT, with the latter weighted more heavily based on recognition of superior accuracy.
